# Designing Dendrimer and Miktoarm Polymer Based Multi-Tasking Nanocarriers for Efficient Medical Therapy

**DOI:** 10.3390/molecules200916987

**Published:** 2015-09-17

**Authors:** Anjali Sharma, Ashok Kakkar

**Affiliations:** Department of Chemistry, McGill University, 801 Sherbrooke St. West, Montreal, QC H3A 0B8, Canada; E-Mail: anjali.25sept@gmail.com

**Keywords:** multifunctional, nanocarriers, dendrimers, miktoarm polymers, nanomedicine, drug delivery

## Abstract

To address current complex health problems, there has been an increasing demand for smart nanocarriers that could perform multiple complimentary biological tasks with high efficacy. This has provoked the design of tailor made nanocarriers, and the scientific community has made tremendous effort in meeting daunting challenges associated with synthetically articulating multiple functions into a single scaffold. Branched and hyper-branched macromolecular architectures have offered opportunities in enabling carriers with capabilities including location, delivery, imaging *etc.* Development of simple and versatile synthetic methodologies for these nanomaterials has been the key in diversifying macromolecule based medical therapy and treatment. This review highlights the advancement from conventional “only one function” to multifunctional nanomedicine. It is achieved by synthetic elaboration of multivalent platforms in miktoarm polymers and dendrimers by physical encapsulation, covalent linking and combinations thereof.

## 1. Introduction

The collective objective of the research community involved in developing efficient therapeutic interventions is to manipulate, wisely engineer, and precisely design nanostructures in terms of their molecular architecture, size, shape, and surface functionalities. It can facilitate their translation from laboratory to clinic in a timely fashion. Nanoscale materials possess a combination of interesting properties which set them apart from bulk systems. In the past couple of decades, there has been tremendous effort devoted to exploiting these unique characteristics to construct a variety of nanomaterials for biomedical applications [1,2,3]. Nanomedicine, an offshoot of nanotechnology, is focused on establishing smart multimodal drug delivery platforms, capable of combining treatment, diagnosis, targeting and monitoring in single nano-scaffold [4,5,6,7,8,9,10,11]. The prospect of resolving key issues when dealing with high morbidity rate diseases including cancer, atherosclerosis *etc.*, has generated increased enthusiasm in the nanomaterials based scientific community [12,13]. It has led to the emergence of various multifunctional nanocarriers as vehicles for the targeted delivery of conventional drugs, proteins, siRNAs, and genes [14,15]. We highlight here a brief summary of the recent progress made in this rapidly evolving field of multifunctional nanostructured materials, specifically focusing on the evolution of dendrimers and miktoarm polymer micelles in this regard.

## 2. Need for Multi-Tasking in Biology

**Figure 1:**
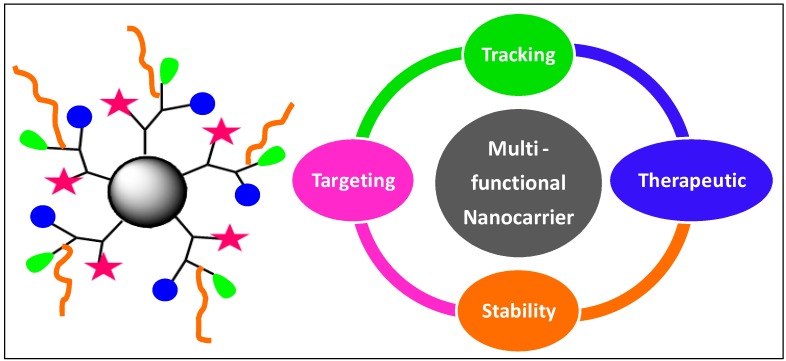
Design of multifunctional nanocarriers for targeted drug delivery.

Medical therapy is enriched with new molecular targets and potent therapeutic agents. Despite showing promising *in vitro* activities, several drug molecules fail to exhibit similar potency in *in vivo*. This could generally be due to the adverse pharmacokinetics and pharmacodynamics of therapeutic molecules, and may include several factors including low bioavailability, non-selective biodistribution, rapid elimination from the body, accumulation in healthy cells, and inability to reach targeted locations. The existing challenge of nanomedicine is to design novel platforms combining drug delivery with diagnostic tools having unprecedented precision and efficacy to obtain maximum treatment payoff. This can be achieved by designing multifunctional nanocarriers, which in addition to the delivery of therapeutic agents, can improve their aqueous solubility, simultaneously target them to the desired intracellular locations, as well as monitor their pathway (Figure 1) [16,17,18,19,20,21]. It is also essential that the circulation and stability of nanocarriers in the blood is sufficient enough to allow their accumulation at the diseased site, and to avoid their degradation in the body prior to reaching their desired destination. Multifunctional nanocarriers can significantly improve the efficacy of therapeutic agents by providing (i) improved aqueous solubility and biodistribution; (ii) extended blood circulation; (iii) enhanced stability; (iv) stimuli responsiveness; (v) active or passive targeting; and (vi) simultaneous monitoring (Table 1). Multi-tasking offers significant potential in expanding the scope of nanomedicine for early detection of fatal diseases with improved therapy and minimal toxicity and side effects [22,23].

**Table 1:** Specific moieties and their functions.

Purpose	Moiety	Function
Targeting [24,25]	Peptides, Antibodies	Specificity and recognition
Imaging agents [26,27]	Dyes, Quantum dots, Magnetic nanoparticles	Monitoring and diagnostics
Stealth [28,29]	Polymers: Polyethyleneglycol, polypeptides *etc.*	Extended blood circulation, aqueous solubility and stability, Biocompatibility
Cell penetrating agents [30,31]	Peptides, cationic polymers, cationic lipids, Transferrin	Enhanced cellular penetration

## 3. Dendrimers and Miktoarm Polymers: Interesting Platforms for Nanomedicine

**Figure 2:**
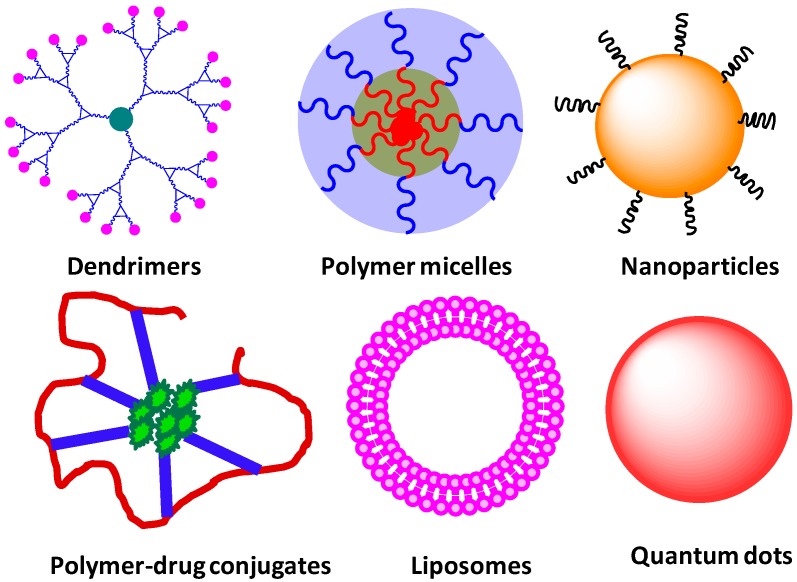
Illustration of different types of nanocarriers for drug delivery applications.

The engineering of multifunctional nanocarriers has offered significant potential in dealing with diseases such as cancer and neurological disorders. This is attributed to the availability of a diverse range of nano-materials, tunable sizes and shapes, surface area, surface functionalization as well as physical incorporation of guests in the interior [32]. Various nano-platforms have been explored for biological applications, and can be divided into categories such as organic, inorganic and hybrid materials (Figure 2) [33]. Organic systems include polymers [34,35,36,37,38], dendrimers [34,39,40,41,42,43,44,45,46,47,48], liposomes [49,50] and carbon nanotubes [51,52], and among inorganics are quantum dots [53,54], silica [55,56] and gold nanoparticles [57]. Hybrid materials are a combination of both organic and inorganic nanoparticles, and include dendrimer entrapped gold nanoparticles [58,59]. Each offers its own features, and the choice is highly dependent upon the desired application. Dendrimers and miktoarm stars are among nanocarriers which have been intensely investigated for drug delivery applications due to their unique characteristics and ease of modification [60].

### 3.1. Dendrimers

Hyperbranched macromolecules commonly referred to as dendrimers [46,48,61,62,63,64,65,66,67,68,69] have well defined molecular architecture which makes them interesting candidates for the development of multifunctional nanocarriers. The synthetic methodologies for their construction can provide precise control of their size, shape, number of end groups and surface functionalities. The term “dendrimer” is a combination of two Greek words *dendron* means “tree” and *meros* means “part”, which exactly reflects their branched architecture [48]. There has been an exponential increase in activity in the field of dendrimers for their rapid and efficient synthesis; and their applications in numerous areas [70] including catalysis [71], electronics [72], sensing [73,74], nanoengineering [75], diagnostics [76], and drug [77] and gene delivery [78]. Some of the most explored dendrimers for drug delivery are poly(amido amine) (PAMAM) [79,80,81,82,83,84], poly(propylene imine) (PPI) [85,86,87,88], poly(l-lysine) (PLL) [89,90,91,92], and triazene [93,94,95] based dendrimers.

**Figure 3:**
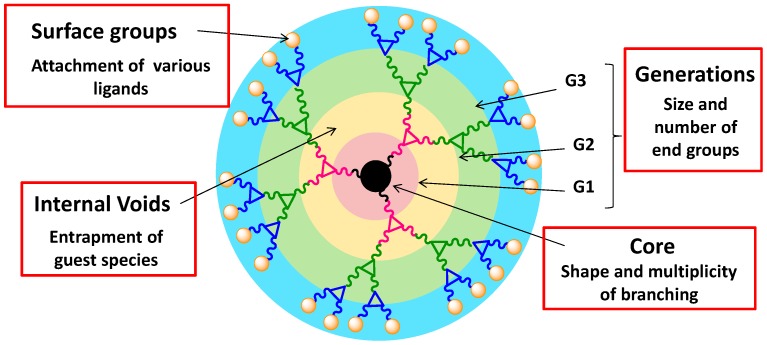
Structural components of a dendrimer.

Dendrimers have a well-defined structure which is composed of different components: (a) central core composed of multiple reactive sites from which branches originate in a symmetric fashion (Figure 3); (b) branching units which are attached in a layer-by-layer fashion giving rise to dendrimer generations, (c) surface end groups which increase exponentially and offer platforms for the covalent attachment of multiple functions; and (d) internal cavities which come into existence after 3rd or 4th generation when dendrimers attain a globular shape. The latter can be used to physically encapsulate guest molecules. The surface functionalities on dendrimers determine the physicochemical as well as the biological properties of the dendrimers. Depending upon the types and nature of surface groups, dendrimers can be hydrophilic or hydrophobic in nature. Moreover, the dendrimer surface also determines their fate for biological applications, e.g., polycationic dendrimers can easily access the cell membranes as compared to neutral ones, but at the same time are more toxic. The elegant synthetic work by numerous groups have helped us fine tune their overall structure to append a well-defined number of multiple functionalities such as therapeutic and imaging agents, targeting ligands and solubilizing entities [18,96,97,98,99,100,101,102,103]. Compared to traditional polymers, dendrimers offer advantages which include for example, (i) low polydispersity; (ii) exact number of surface end groups; (iii) tailor made structure; (iv) controlled size and shape; (v) ability to covalently attach or physically sequester drug molecules and ligands; (vi) multiple attachment sites, and (vii) efficient cellular uptake.

From basic fundamental research, dendrimer chemistry is now translating into applied research extensively in the field of nanomedicine. Dendrimers have features which make them excellent candidates for the development of multifunctional drug delivery systems [47,48]. The size of the dendrimers is small enough (e.g., PAMAM dendrimers are in the range of 1–10 nm) enabling their clearance through renal filtration, thus, eliminating the need to design them to be biodegradable [104]. Unlike traditional polymers, dendrimers are generally synthesized in a step-by-step iterative fashion providing precise control, and assuring reproducibility of the pharmacokinetic behavior of the nanocarriers. The polydisperse nature of traditional polymers has posed serious rejections in clinical trials due to their non-reproducible pharmacokinetic profiles. Multivalent periphery of dendrimers can be conveniently utilized to append conventional drug molecules, targeting agents and imaging moieties by covalent conjugation. For biological applications, the exact dosage of the drug or the payload is of utmost importance. Dendrimers provide this opportunity, and the presence of multiple bioactive molecules together at the surface of single nanoscaffold leads to several folds enhancement in the therapeutic efficiency, which is also known as “synergistic” or “multivalent” effect [105,106]. Alternatively, the interior of the dendrimers with well-defined cavities can also be adopted as a host to incorporate various therapeutic agents through non-covalent interactions (e.g., ionic, hydrophobic, and hydrogen-bond interactions) [107,108]. Both surface and internal cavities of the dendrimers can be simultaneously utilized to develop multifunctional systems [109]. The core of the dendrimers itself can serve as a template for a functional moiety, and the surface groups of dendrimers can be altered according to biological needs [110]. For example, the attachment of solubilizing polyethylene glycol at the periphery can extend the circulation time of nanocarriers in the blood and avoid opsonization. Thus, it provides an opportunity to accumulate at pathological sites. In addition, the peripheral groups can also be modified to reduce toxicity or for biocompatibility. It is highly desirable for the nanocarriers to deliver the drug at the diseased area. Several strategies are being employed for this purpose including attaching the drugs to the nanocarriers along with the targeting moieties. Recently, much effort has been devoted to developing stimuli responsive nanocarriers, which can deliver their payload in desired cellular environments, thus avoiding their accumulation in healthy tissues [111]. This strategy is particularly useful for the treatment of diseases such as cancer. Dendrimers offer versatile scaffolds for grafting different functional moieties on the periphery, in the interior, or even at the core thus, paving a way towards the design of “smart” nanomaterials for successful and efficient medical therapy.

**Figure 4:**
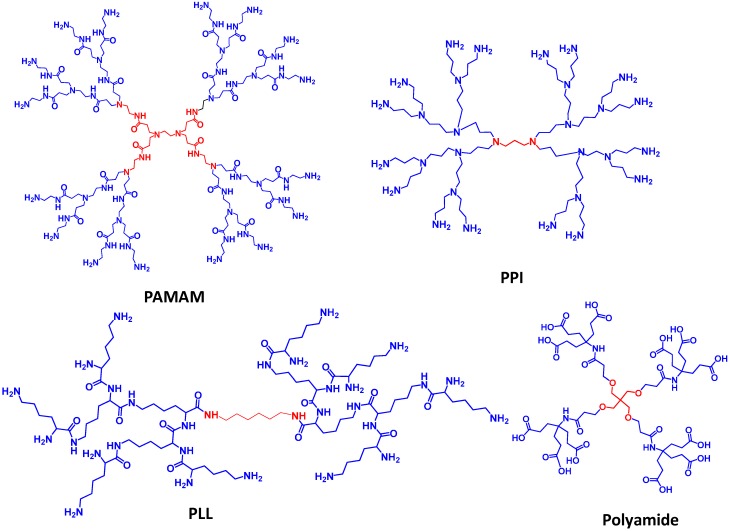
Structures of dendrimers utilized in biological applications.

Many dendrimers are now commercially available, and have gained success up to the advanced phases of clinical trials. The structures of some commonly used dendrimers for drug delivery applications are presented in Figure 4. Dendrimer based anti-HIV drug VivaGel^®^ has been developed by Australian company Starpharma, and is under clinical evaluations as an active ingredient in a vaginal microbicide gel, and for coating condoms as potential contraceptive due to its spermicidal properties [112]. The active scaffold of the drug is a fourth generation poly lysine dendrimer. OcuSeal^®^ (Beaver-Visitec International), a hydrogel-dendrimer liquid ocular bandage is an effective microbial barrier to block bacterial penetration while stabilizing ocular wounds following surgical or non-surgical trauma [113]. Gadomer-17 is a polylysine dendrimer based contrast agent with 24 gadolinium-DOTA complexes which is being investigated as magnetic resonance contrast agent [114]. One more dendrimer based commercial product, Stratus CS, Acute Care Diagnostic System from Siemens, is a cardiac biomarker which uses polyamidoamine (PAMAM) dendrimers based assay technology and provides rapid and early detection of myocardial ischemia [115]. “Alert Ticket” is a dendrimer based diagnostic agent developed by US army research laboratories being used for Anthrax detection. Qiagen has developed a polycationic dendrimer based transfection material for *in vitro* DNA transfection [116]. The cationic groups on the dendrimer can make complexes with negatively charged phosphates of nucleic acids, called dendriplexes.

### 3.2. Miktoarm Star Polymers

**Figure 5:**
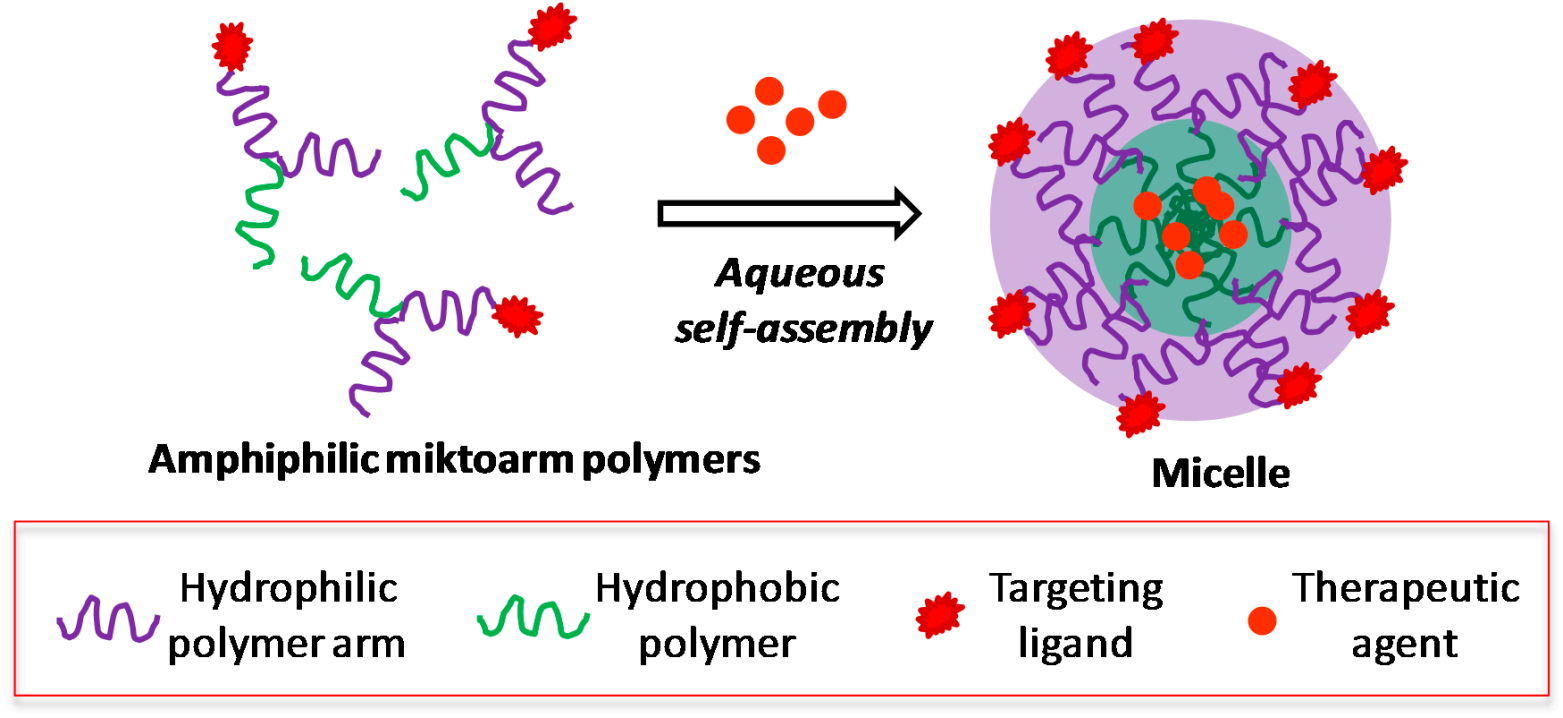
Aqueous self-assembly of amphiphilic miktoarm polymers with entrapped drug molecules.

Polymeric micelles are the most extensively studied supramolecular architectures for biomedical applications including the delivery of therapeutic agents, genes, proteins and DNA *etc.* [37] Instability of micelles from diblock linear polymers can lead to premature drug release. As discussed earlier, multi-tasking is one of the key tenets of nanomedicine, and it requires nanocarriers to simultaneously perform multiple functions including drug loading and delivery, avoiding premature drug release, monitoring the fate of the drug as well as the carrier, and targeting the nanocarriers to intended site of action. In this regard, amphiphilic miktoarm star polymers are of specific interest to design synthetically articulated and tailor made micelles, and are currently being investigated extensively for the development of multifunctional nanocarriers for biological applications [117,118,119,120,121]. Miktoarm polymers have star shaped well-defined molecular architecture where different polymeric arms originate from a common central core. The polymeric arms may differ in the type of polymers or may constitute similar polymers of different chain lengths. The supramolecular self-assembly of amphiphilic miktoarm star polymers from aqueous solutions generates micelles having unique core/corona type architecture (Figure 5). The hydrophilic polymeric segments of the miktoarm stars contribute to the construction of outer corona of the micelles, while hydrophobic polymeric chains constitute the core of the micelles. The hydrophobic interior of the micelles serves as a nanocontainer for the encapsulation of hydrophobic drugs increasing their aqueous solubility, and the outer corona helps in stabilization of the supramolecular structure, and at the same time, it could act as a platform to covalently link the functional moieties.

Miktoarm polymers offer several benefits when compared to traditional linear polymers for developing multifunctional nanocarriers in performing integrated functions. Moreover, micelles can be rationally designed to be stimuli sensitive to release drugs at the targeted site [122]. Although micelles obtained from miktoarm polymers may have similar topological structures to those from linear polymers, they can undoubtedly produce more stable aggregated morphologies in selected solvents. Miktoarm polymer micelles have been demonstrated to provide higher drug loading, better delivery efficiency, lower critical micelle concentrations (CMCs), narrow size distribution, and a controlled sophisticated synthesis [60,117]. In addition, owing to multiple polymeric arms, a variety of functions can be introduced at the periphery of the micelles which is extremely difficult and rare with traditional linear polymers. This includes conjugation of targeting moieties for enhancing intracellular uptake, and fluorescent dyes for providing tracking capabilities. Both the surface and the interior of miktoarm polymer micelles can be synthetically fine-tuned according to the need of a specific delivery platform. 

## 4. Monofunctional to Multifunctional Nanocarriers: Synthetic Challenges

### 4.1. Dendrimers

#### 4.1.1. Dendrimer Synthesis

The development of highly efficient and rapid synthetic methodologies in combination with the design of novel orthogonal building blocks have provided precise control over the synthesis of multifunctional branched and hyper-branched macromolecules. Traditionally, dendrimers were synthesized using classical divergent and convergent approaches, which differ in the directionality of dendrimer growth. The divergent synthesis initiated by Tomalia, Newkome, and Vögtle is accomplished starting from the core towards the periphery, and thus is an inside-outward approach [48,123]. The convergent methodology involves the construction of dendrimers from outside-in, in which dendrons are synthesized first, and are subsequently coupled to a branched core [124]. Both methods have their own pros and cons, as with an increase in generation of dendrimers using divergent growth, the number of reactive end groups at the periphery increases exponentially. It becomes extremely difficult then to obtain 100% conversion to get defect free dendrimers at higher generations. The purification of dendrimers becomes cumbersome, and in addition, an excess of reagents is required to carry out conversion on multiple end groups. The benefit of using divergent methodology is that the synthesis can be controlled and stopped at any stage according to the need of number of end groups and the size of dendrimers. The classical divergent route provides functional groups with similar relativities at the ends. However, the combination of divergent strategy with orthogonal approaches have somewhat overcome this hurdle. The convergent approach, on the other hand, can generate defect-free dendrimers due to the presence of less number of terminal reactive groups. As the dendrons are smaller in size, the purification is comparatively easier.

#### 4.1.2. Accelerated Approaches for Dendrimer Synthesis

To meet the ever-growing demand of dendrimers for a variety of applications, a significant effort has been devoted to developing accelerated approaches for dendrimer synthesis, by modifying or employing a combination of classical methods. The primary focus was to develop defect-free dendrimers in minimum reaction steps making the synthesis rapid, and cost-effective. The aim of highly efficient and robust chemical reactions or one-pot synthesis has been to accelerate the synthesis in order to develop high generation dendrimers in fewer steps. The dendrimer synthesis is continuously improving and a number of accelerated approaches, which include hyper-monomer strategy, double stage convergent, double exponential growth, one pot synthesis, multicomponent reactions, and onion peel, have been reported [69,125,126,127,128,129,130]. The accelerated synthesis has also been used for the introduction of multiple functions, for example the double stage convergent methodology, which allows placing different groups internally and on the surface of dendrimers, thus giving rise to a block dendrimer.

#### 4.1.3. The “Click” Chemistry and Orthogonal Strategies

**Figure 6:**
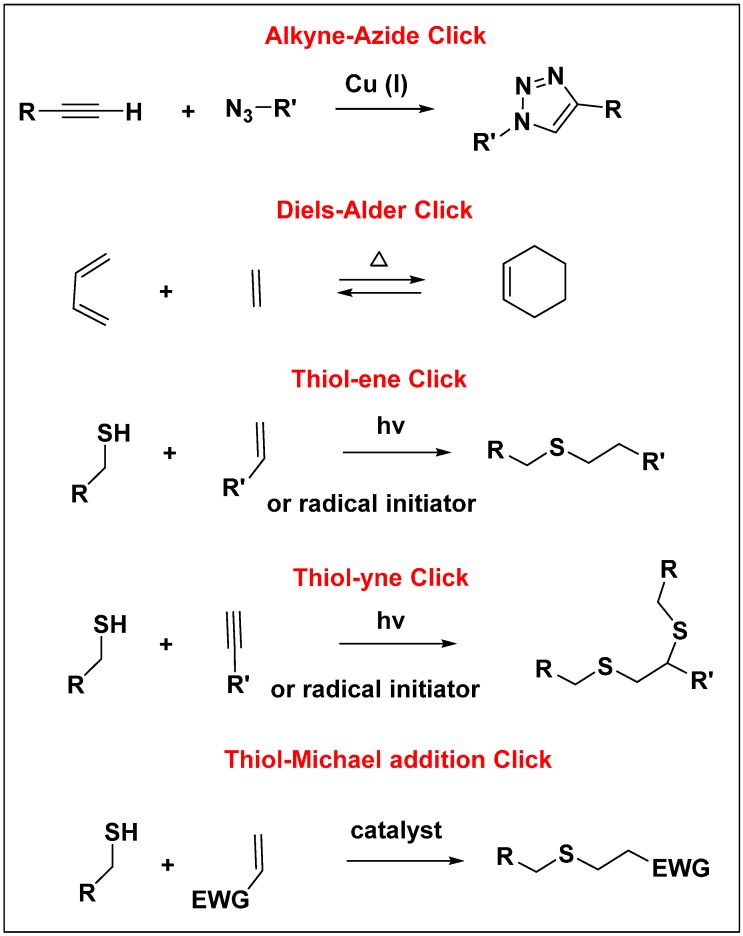
Different types of click reactions.

The click chemistry concept was first reported by Sharpless in 2001, and since then has emerged as an effective synthetic tool to build a large variety of small as well as macromolecules [131]. The click reaction is associated with high yields, minimum side products, efficient reaction rates, and tolerance to a variety of functional groups and solvents. The commonly studied click reactions include, Cu(I) catalyzed alkyne-azide (CuAAC), thiol-ene, thiol-yne, thiol-Michael addition, and Diels-Alder reactions (Figure 6).

CuAAc is the most widely used click reaction so far, and it was introduced to dendrimer synthesis in 2004 [132]. Since then, it has been employed to synthesize dendrimers in both convergent and divergent ways, to functionalize their periphery, to connect different dendrons together creating multi-block dendrimers, and to develop multi-functional dendrimers using orthogonal building blocks [133,134,135,136,137,138,139]. The complete removal of residual copper has been challenging, and could pose a problem for dendrimers meant for biological applications. An alternative in which copper free strain promoted click reaction using alkynes such as cyclo-octyne, has been developed [140]. It has not been extensively accepted though, as it introduces the unwanted cyclo-octyne rings into the structure of dendrimers, which brings additional issues for applications in biology.

The introduction of “click” chemistry to the field of dendrimers has dramatically improved the synthesis by combining it with orthogonal approaches. One can drastically reduce the number of reaction steps by using building blocks able to undergo orthogonal coupling reactions, which eliminates the need of protection/deprotection steps throughout the synthesis. Malkoch *et.al.* have developed an elegant approach to construct fourth generation bis-MPA and Fréchet type dendrimers [141], and have also demonstrated the synthesis of a sixth generation dendrimer starting from its monomers in a single day [142].

#### 4.1.4. Heterofunctional/Multifunctional Dendrimers

**Figure 7:**
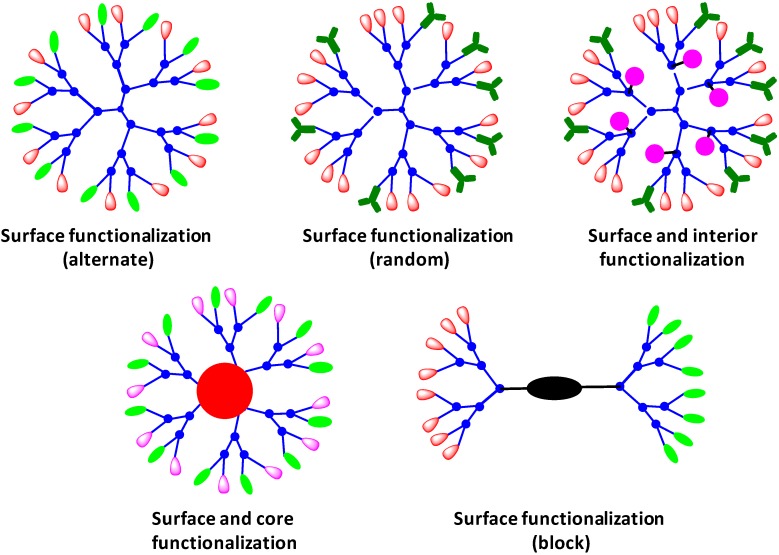
Surface, interior and core functionalized heterofunctional/multifunctional dendrimers.

Much of the emphasis on the work reported on dendrimers has been done on monofunctional systems, and the strategies to introduce multiple functions into a single dendrimer are quite limited. The ideal dendrimer based drug delivery system needs to accommodate different functions in to a single carrier. Multiple functions can be introduced into dendrimers in a variety of ways using their periphery, core, as well as the interior (Figure 7). Due to the similarity in reactivity of surface groups in a monofunctional dendrimer, it is tedious to incorporate different functions using a post-synthesis approach. Most widely explored PAMAM dendrimers for drug delivery applications have been functionalized using a random statistical approach by partial functionalization of terminal amines. It is hard to control the reproducibility using such strategies and requires multiple steps to achieve dendrimers with well-defined multiple end groups [143]. Kannan and coworkers have reported heterofunctional PAMAM dendrimers generated through simple one-step synthetic methodology by nearly complete end-capping (87%–93%) of G4 PAMAM dendrimers [144]. The application in drug delivery requires the information about exact number of drug molecules to estimate the dosage, and in the case of random statistical functionalization, only an estimation of the conjugation can be calculated using analytical characterization techniques. Thus, it is highly desirable to develop synthetic strategies which can introduce multiple desired functions in a controlled fashion.

Heterofunctional dendrimers provide tools to develop well-defined multifunctional nanocarriers for drug delivery applications. There are only a few reports of true heterofunctional dendrimers, and the reports where more than two functions have been successfully introduced in a single dendrimer scaffold are still rare in the literature. There can be different arrangements of functional moieties in the interior and exterior of the dendrimers, giving rise to a variety of heterofunctional dendrimers. The dendrimers can have different functional units in block, alternating or random manner [69]. The synthesis of a trifunctional poly(amide)-based dendrimer containing 16 protected acid groups, one azide and one aldehyde group has been reported by Weck and coworkers [145]. The dendrimer was synthesized around a trifunctional core which could be monofunctionalized selectively. Hawker and coworkers have reported an elegant methodology to covalently attach molecules in the interior of the dendrimer, keeping the orthogonal surface for attaching other functions [102]. The covalent encapsulation of the drug in these systems could provide better control in its stability as well as release. We have presented a versatile strategy to synthetic tools which can be easily adapted to construct a variety of multifunctional dendrimers with different combinations of drug, imaging agents and solubilizing polymers. A series of bi- and trifunctional dendrimers were synthesized with well-defined compositions of peripheral functions using orthogonal building blocks in combination with click chemistry [99,100]. We have also successfully engineered both the core and the periphery of the dendrimers to incorporate multiple functions, and have reported inherently fluorescent multifunctional traceable dendrimers for visualizing drug delivery [98]. More recently, Zimmerman’s group has published a water-soluble polyglycerol dendrimer with core having two orthogonal functional groups, an azide and an amine [146]. The orthogonal functionalization could be achieved in step wise or one-pot method giving rise to water soluble bifunctional dendrimers with 48 hydroxyl groups on the surface which could successfully solubilize the hydrophobic fluorophore and targeting moiety. Although the list of truly multifunctional dendrimers is not vast, the continuous progress is being made in the development of efficient, accelerated, one-pot and orthogonal chemistries for the synthesis of structurally perfect, reproducible and sophisticated multifunctional dendrimers a success.

### 4.2. Multifunctional Miktoarm Polymer Micelles

Miktoarm star polymers have recently gained significant interest among amphiphilic block co-polymers due to their unique architecture and ease in synthetic articulation. The presence of multiple polymeric chains makes star polymers excellent candidates to develop multifunctional nanocarriers. The ability to introduce various functions into miktoarm polymeric micelles significantly enhances their potential for biomedical applications. The use of pH responsive or temperature responsive polymeric segments is another interesting approach to promote intracellular drug release by the application of internal or external stimuli. Zhou and coworkers have developed a multifunctional micellar system with a combination of active targeting capability and redox-responsiveness [147]. *In vivo* studies suggested that the multifunctional redox-responsive star polymer micelles produced a better therapeutic effect to artificial solid tumors and provided higher safety to healthy tissues as compared to redox-insensitive micelles.

**Figure 8:**
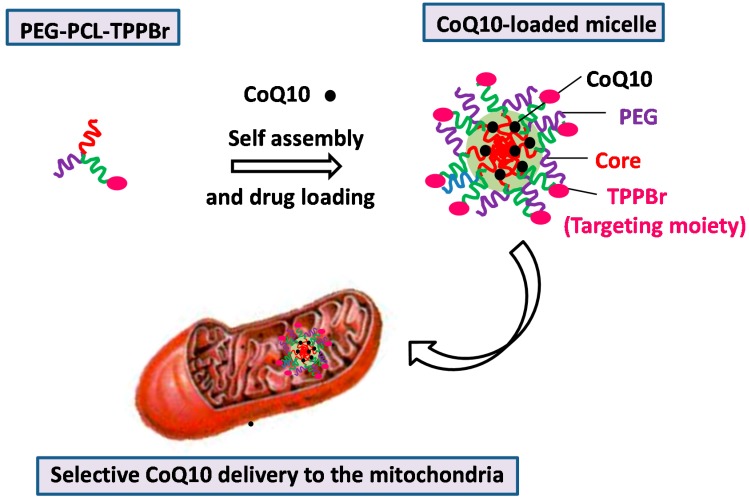
Organelle specific drug delivery to target mitochondria using multifunctional self-assembled ABC miktoarm star micelles.

Organelle specific drug delivery presents a highly sought after goal in biology. Our group has recently reported a versatile and convenient methodology to construct multifunctional nanosystems based on ABC miktoarm star polymers using a combination of click chemistry and ring opening polymerization to target mitochondria (Figure 8) [148]. Mitochondrial impairment can cause the failure of various cellular functions leading to different pathologies. The star polymer micelles were self-assembled from ABC miktoarm stars comprising of PEG as a hydrophilic segment, polycaprolactone (PCL) as a hydrophobic polymer and triphenylphosphinium bromide (TPPBr) acting as a targeting ligand for mitochondria. The studies demonstrated that the miktoarm micelles had the ability to incorporate large quantities of coenzyme CoQ10 (ubiquinone) compared to other polymeric systems, and were able to reach mitochondria. This strategy can be easily elaborated to develop various site-specific drug delivery systems of interest using a combination of different therapeutic molecules and targeting moieties.

The concept of combined therapeutics with diagnosis (theranostics) has recently become an integrated part of drug delivery systems, specifically for the treatment of high morbidity diseases such as cancer [149]. The strategies are being designed to develop nanocarriers which can simultaneously deliver cargo molecules and have tracking capabilities as well. In order to achieve these multifunctional systems, there is a need to create smart polymeric materials with highly reactive chain ends, which can be further used to articulate different varieties of bioactive molecules and imaging moieties. Lu and coworkers have reported the design and synthesis of well-defined multifunctional star polymers with highly reactive and thermoresponsive poly(NIPAM-co-acrolein) arms, and a fluorescently labeled core bearing aluminum tris(8-hydroxyquinoline) (Alq3) [150]. The polymer was successfully synthesized via reversible addition-fragmentation chain transfer (RAFT) polymerization reaction using “arm-first” technique. The presence of highly reactive aldehyde groups in the arms of star polymer could be easily used to conjugate a variety of molecules of interest for different biological applications. The star polymer demonstrated a lower critical solution temperature and showed intense greenish-yellow fluorescence with an emission maximum around 520 nm in both organic solvents and water. Our group has recently reported an approach to synthesize inherently fluorescent traceable multifunctional nanodelivery system developed from self-assembled ABC miktoarm star polymers containing PEG, PCL and covalently attached tetraiodofluorescene dye [151]. The star polymers self-assembled from aqueous solutions into micelles incorporating an imaging probe at the center. We demonstrated that the inherently fluorescent micelles with the capability of tracing them in live cells could significantly enhance the solubility, loading, and sustained release profile of hydrophobic drugs like curcumin. Such miktoarm star polymers have proven potential for the development of multifunctional self-assembled nanostructures to deliver drugs with poor pharmacokinetic properties, while their inherent fluorescence capability can help determine their fate.

## 5. Covalent Conjugation and Non-Covalent Encapsulation in Designing Multifunctional Nanocarriers

### 5.1. Covalent Conjugation of Drugs and Bioactive Ligands to Dendrimer Surfaces

The presence of ample surface groups at the periphery of dendrimers provides a platform for the chemical conjugation of drug molecules with suitable functional groups. The attachment of multiple copies of same therapeutic agent on a single nanocarrier results in enhancement of therapeutic response due to synergistic or multivalency effect. In addition, the attachment of targeting agents and solubilizing polymers along with the bioactive molecules can even further improve the pharmacokinetic profile. The drug bound to nanocarrier diffuses slower than the free drug, thus, increasing its blood circulation time providing an opportunity to interact with targeting tissues. Dendrimers can be synthetically articulated to carry a well-defined payload of therapeutic molecules. Although physical encapsulation of drug molecules in the dendrimers is possible, but only a few molecules of drugs can be encapsulated even in the case of high generation dendrimers with well-defined internal cavities. Furthermore, the covalent attachment of the drug molecules provides better control in terms of their release which can only take place through physiological trigger either by chemical or enzymatic cleavages of the bonds attaching the drugs to the nanocarriers [152]. The drugs can be appended on the surface of dendrimers using a variety of chemical linkages, and most commonly employed are ester or amide bonds, but in addition to these the drugs can also be appended using peptide, disulfide, carbamate and hydrazone linkages [153,154]. Different chemical bonds have their different mechanisms of cleavage, for example, ester, hydrazine and carbamate linkages are pH dependent, while peptide and amide bonds undergo enzymatic cleavages. Disulfide bonds are glutathione sensitive and can be reduced in cytosol [155]. Several drugs have been successfully conjugated to the multivalent surface of PAMAM dendrimers, e.g., naproxen, 5-aminosalicylic acid, venlafaxine [156,157]. The studies have shown that the dendrimer–drug conjugates exhibit enhanced solubility and controlled release as compared to their free drug counterparts.

Well-defined distribution and precise attachment of drug molecules at the surface of dendrimers are highly crucial factors to consider while developing a drug-delivery system. PAMAM dendrimers are the most widely studied for drug delivery applications as they conform to most of the requirements for *in vivo* studies [158,159]. The drugs are often conjugated to the surface of PAMAM dendrimers using a random statistical approach. Due to the presence of a large number of end groups with same reactivity and growing surface compactness at each generation, it becomes highly tedious and unavoidable to control the exact number and position of drug molecules being anchored at the periphery. This results in the loss of well-defined, precise and monodisperse characteristics of dendrimers and makes them heterogeneous structures. Moreover, isolation and analyses of these heterogeneous mixtures of dendrimer–drug conjugates becomes extremely difficult due to similarity in their solubility and properties. Different dendrimer–drug conjugates with variable number of therapeutic molecules differ in their pharmacokinetic profile. Structural heterogeneity of dendrimer–drug complexes is a major hurdle towards their translation from lab to clinic [104]. Recently, Murdoch and coworkers have reported molecularly-precise dendrimer–drug conjugates with tunable drug release for the treatment of cancer [160]. Instead of utilizing the periphery of dendrimers where it is nearly impossible to control the number and position of attachment of drug molecules, they used camptothecin molecule as the core of poly(l-lysine) dendrimer and the resulting dendrimer–drug conjugate had precise molecular structure with fixed drug content. Furthermore, the release rates of drug from these conjugates were tunable and were dependent on dendrimer generation, surface chemistry and acidity.

The problem of heterogeneity during the synthesis of dendrimer–drug conjugates becomes even worse while designing multifunctional systems, where together with the drug molecules, targeting ligands and imaging agents are to be attached at the periphery. The addition of each of these ligands further results in heterogeneous mixture of nanocarriers with batch to batch inconsistencies and variable biological responses. In order to address this issue, there is a need to develop efficient synthetic strategies which can allow the incorporation of a defined number of therapeutic molecules, and other bioactive ligands including agents for targeting, imaging *etc.* There are few examples in literature where monodisperse multifunctional dendrimers with precise number of attached ligands have been developed for biological applications. For instance, Baker’s group had reported a multifunctional G5 PAMAM dendrimer based nanocarrier for targeted cancer therapeutics, containing covalently conjugated folic acid (FA) as cancer targeting agent and methotrexate (MTX) as a chemotherapeutic drug [161]. Although the nanocarriers were able to selectively target and kill cancer cells in *in vivo* and *in vitro* studies, the synthetic strategy used to construct conjugates resulted in heterogeneous mixtures with variable numbers and ratios of FA and MTX [161]. The batches were even more inconsistence for large scale synthesis. In order to resolve this issue, recently Baker’s group developed a triazine scaffold approach which can not only reduce the number of synthetic steps for conjugation, but can produce G5 conjugates with defined ratios of FA and MTX [143].

**Figure 9:**
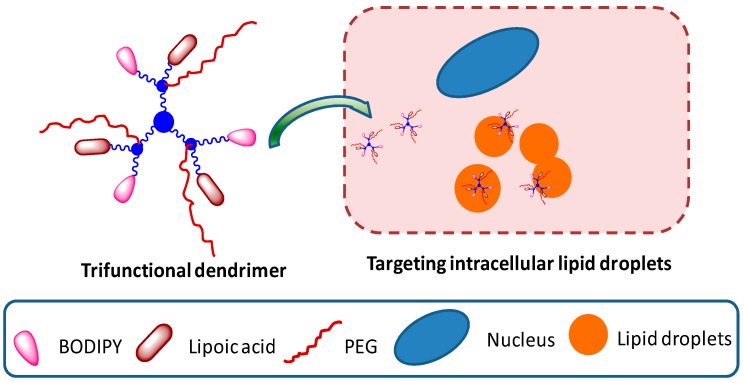
A trifunctional dendrimer targeting intracellular lipid droplets.

Our group has reported a simple, versatile and most importantly a general synthetic approach to covalently graft precise number of drugs, imaging dyes, and solubilizing polymers at the dendrimer surface [99]. The methodology is based on employing orthogonal building blocks stitched together using click chemistry and Steglich esterification to develop well-defined multifunctional nanoplatforms. This methodology easily allows the fine-tuning of dendrimer structure to attach desired combination and numbers of functional ligands at the surface. We have successfully demonstrated the synthesis of a variety of bi-functional and a tri-functional dendrimer having α-lipoic acid as a model drug, BODIPY dye as an imaging agent, and PEG as solubilizing polymer (Figure 9) [99]. The dendrimers were noncytotoxic within micromolar concentration, were able to target intracellular lipid droplets, and were effective in reducing reactive oxygen species in pheochromocytoma cells.

**Figure 10:**
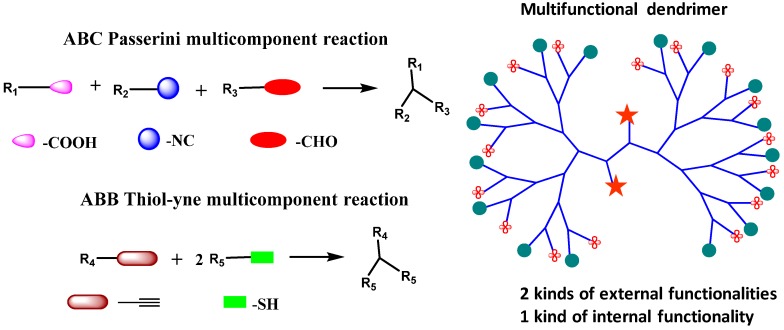
Multifunctional dendrimer with two types of external functional group and one type of internal functional group synthesized by a combination of ABC Passerini and ABB thiol-yne MCR reactions.

Another approach is to use highly efficient and orthogonal multicomponent reactions to introduce a defined number of multiple end-groups in dendrimer structure which can be utilized further for bioconjugation. Most recently, Li and coworkers demonstrated an efficient method to synthesize dendrimers with precise multiple functions [130]. Generation 2 dendrimer was synthesized by a divergent approach using a combination of orthogonal ABC Passerini multicomponent reaction (MCR) and ABB thiol-yne MCR containing one kind of internal functional group and two kinds of surface functional moieties in five steps (Figure 10). The final dendrimer structure had three different kinds of functional groups in a controlled and fixed arrangement.

The covalent surface conjugation of drugs provides several advantages over physical encapsulation, for instance, covalent conjugation can slow down the premature drug release, and can allow the design of stimuli sensitive targeted drug delivery systems. The surface bio-conjugation is associated with some drawbacks which cannot be ignored. The presence of a large number of drug or bioactive molecules at the surface alters the properties of dendrimers, reducing their solubility as well as biocompatibility. This problem can somehow be resolved by attaching solubilizing and biocompatible polymers at the surface which can help in increasing the blood circulation time of the nanocarriers. Another issue with surface conjugation of drugs is the direct contact of the drugs with enzymes and body fluids, while the drug encapsulated in the interior of nanocarriers remains protected. Some researchers are working towards combining the advantages of both surface functionalization and interior non-covalent encapsulation. A strategy to develop internally but covalently functionalized dendrimers has been reported by Hawker’s group by combining epoxy-amine and thiol-ene click chemistry, to introduce reactive hydroxyl groups at each layer of dendritic growth [162].

### 5.2. Physical Entrapment of Drugs within Dendrimers and Star Polymer Micelles

#### 5.2.1. Drug Encapsulation within Dendrimers

The grafting of the drugs by covalent attachment requires the alteration of chemical structures of both the drug as well as dendrimers which makes the molecular design a complex process. In addition, a change in the structure of therapeutic molecule can decrease its biological activity. In order to simplify these issues, direct entrapment of drug molecules can be carried out inside the dendrimers or polymer micelles. The advantage of using dendrimers over polymeric micelles as carriers for physical encapsulation is that these nanocarriers do not disintegrate upon dilution in systemic circulation and, thus, do not cause the premature drug release. The basic requirement for physical encapsulation is the presence of hydrophobic interior with well-defined internal cavities or supramolecular sites in the dendrimer structure and a hydrophilic dense outer shell. However, several challenges are associated with the synthesis of high generation dendrimers, as it generally consists of multiple steps with tedious purifications. Moreover, it is troublesome to synthesize a defect-free dendrimer at higher generation due to multiple reactions involved at the surface and steric hindrances. In the absence of a highly dense peripheral shell, the guest molecules can easily diffuse in and out of the dendrimer. PAMAM and PPI dendrimers have been extensively utilized as hosts for the physical entrapment of drug molecules due to the advantage of internal tertiary amines able to participate in hydrogen bonding interactions with guest species [163]. The encapsulation efficiency of these dendrimers varies with their size, structure, hydrophilic/hydrophobic properties and length of the repeating units [164]. The presence of surface and internal amines in the dendrimer structure can interact with the weakly acidic guest molecules through electrostatic interactions, while the hydroxyl groups in the dendrimer structure can facilitate the encapsulation of drugs through hydrogen bonding. In addition, several other factors affect the encapsulation efficiency including the size and structure of drug molecules and pH of the solvent. At several instances, the dendrimer surface has been functionalized with PEG chains to create a hydrophilic shell in order to enhance the solubility of hydrophobic drugs encapsulated in the interior of the dendrimers. For example, PAMAM dendrimers conjugated with PEG chains of varying lengths have been studied for the encapsulation of anticancer drugs, adriamycin and methotrexate [108]. PEG 500 or 2000 was grafted onto G3 or G4 PAMAM dendrimers. The encapsulation efficiency of these dendrimers increased with increase in generation number of the dendrimers and the chain length of PEG grafts. Maximum encapsulation was observed with G4 PAMAM conjugated with PEG 2000, which could hold 6.5 adriamycin molecules or 26 methotrexate molecules for each dendrimer.

Physical encapsulation of drugs inside the dendrimers has been well documented to enhance the water solubility of several hydrophobic drug molecules including anticancer, anti-microbial and anti-inflammatory agents [165]. By encapsulating the drugs within the dendrimers, the surface groups provide additional room for the attachment of other bioactive materials including targeting ligands, imaging dyes or PEG chains. Recently, Multifunctional lactobionic acid (LA) modified PAMAM dendrimers based targeted drug delivery system has been reported for the treatment of liver cancer cells having overexpressed asialo glycoprotein receptors (ASGPR) [109]. Generation 5 PAMAM dendrimers were modified with fluorescein isothiocyanate and LA (with and without PEG spacer), followed by the acetylation of remaining terminal amine groups. A model anticancer drug, Doxorubicin (DOX), was encapsulated. The conjugation of LA as the targeting ligand provided specific targeting of dendrimer/drug complexes to cancer cells as compared to the non-targeted dendrimer/DOX complexes. Importantly, the presence of PEG spacer between LA and the dendrimer carrier improved anti-tumor therapeutic efficacy of the drug by providing relatively faster release and enhanced target specificity to ASGPR overexpressing liver cancer cells.

Physical encapsulation of drugs in the interior and chemical conjugation at the surface of dendrimers have their own pros and cons and a few review articles have discussed these in detail [104,164]. A lot of reports using dendrimers for drug delivery applications describe these two strategies independently, however, not many researchers have evaluated the effect of physical encapsulation and chemical conjugation on same dendrimer platform simultaneously. Baker’s group has compared the efficacy of G5 PAMAM dendrimers for targeted delivery of MTX using both approaches [166]. Folic acid was conjugated to the dendrimer as a cancer targeting ligand. MTX was covalently conjugated through ester linkage and the cytotoxicity was compared to physically encapsulated MTX. They demonstrated that while the dendrimer-drug inclusion complex is quickly released and active *in vitro*, the covalent conjugation of the drug provided better target specificity.

#### 5.2.2. Drug Encapsulation within Miktoarm Star Polymer Micelles

A wide variety of polymeric micelles have been studied for drug delivery applications and among them micelles obtained from amphiphilic miktoarm star polymers provide an effective way to design targeted drug delivery systems. Miktoarm stars can be used for the attachment of functionalities including targeting and imaging agents, while hydrophobic drugs can be loaded into the interior of the micelles. The advantage with the star polymer micelles for cancer therapy is that the drug delivery system can make use of both active transport mediated by targeting ligands and passive transport controlled by enhanced permeability and retention effect, a unique characteristic of tumor tissues. Unlike dendrimers, polymeric micelles have metastable character due to which they can easily disintegrate in systemic circulation upon dilution. This disintegration can be delayed by the careful choice of polymeric segments while designing the star polymers which can prolong their blood circulation time, modify the drug release profile and make them sufficiently stable for biological applications. The hydrophilic corona can be composed of polyethers like PEG chains of variable chain lengths, provide stealth properties and avoid the uptake by reticuloendothelial system (RES). On the other hand, the hydrophobic core can contain polyesters and poly(l-aminoacids) *etc.* The terminal functional groups of these polymers can be utilized to append other moieties making the micelles multifunctional. The length of hydrophobic polymer also controls the critical micelle concentration (CMC), which increases with increase in the chain length of hydrophobic polymer, but a change in the length of hydrophilic polymer segment does not seem to have any significant effect on CMC [60,167].

Miktoarm polymer micelles have a unique architecture which offers opportunities to fine tune their overall structure by carefully designing the chemistry of polymers comprising the core and the corona. As compared to linear block copolymers, miktoarm star polymers have better drug loading capacity and can reduce the CMC. The drug loading efficiency of polymer micelles is dependent on the compatibility between the drug and the hydrophobic polymer chain, and can be estimated using Flory-Huggins interaction parameter [168]. Our group has reported an A_2_B type miktoarm polymers based nanocarriers for the delivery of nimodipine (NIM), a poorly water soluble hydrophobic drug [117]. The polymers consisting of two segments of PEG and one PCL arm were synthesized using a combination of click chemistry and ring opening polymerization. The micelles obtained from these star polymers showed NIM encapsulation efficiency of up to 78 wt%, and were able to increase the aqueous solubility of NIM by ~200 fold. In addition, the drug followed a sustained release profile and remained protected from precipitation in physiological medium. Recently, we have used similar polymers for the encapsulation of curcumin to treat glioblastoma multiform tumor using combination therapy. Curcumin has poor aqueous solubility and low bioavailability [121]. Micellar encapsulation significantly improved the solubility of curcumin, and the combination of curcumin loaded micelles with pifitrin and temozolamide were quite effective in killing glioblastoma cells.

A continuous effort is being made to effectively design drug delivery systems with improved therapeutic efficacy and minimum side effects. Recently, the research is focused on developing stimuli-sensitive nanomaterials, which can respond to specific internal or external environmental fluctuations and trigger the drug release. A variety of stimuli have been studied for drug delivery applications including, pH, temperature, light, enzymes, and redox [169]. For instance, Zhang and coworkers have reported the design and synthesis of amphiphilic miktoarm star copolymer poly(ε-caprolactone)_3_-[poly(2-(diethylamino)ethyl methacrylate)-*b*-poly(poly(ethylene glycol) methyl ether methacrylate)]_3_ [(PCL)_3_-(PDEAEMA-b-PPEGMA)_3_] as pH sensitive micelles for the delivery of DOX [120]. The studies demonstrated that a decrease in pH from 7.4 to 5.0 could cause the protonation of tertiary amine groups of DEAEMA leading to globule-uneven-extended conformational transitions of micelles, significantly accelerating the drug release rate. Zhou *et al.* has designed and reported a multifunctional star-shaped micellar system combining active targeting ability and redox responsive behavior to deliver the drug to the solid tumor with high therapeutic efficiency with minimum exposure to healthy tissues [147]. The micelles were self-assembled from four-arm PEG-PCL copolymer and anti-cancer drug DOX was entrapped within the micelles during the self-assembly process. The hydrophilic and hydrophobic polymer chains were connected together with redox sensitive disulfide bridges. Cancer targeting ligand FA was appended on the terminal of PEG chain to provide active targeting. *In vivo* studies demonstrated that these active-targeting and redox responsive multifunctional micelles possessed better antitumor activity and higher safety to normal tissues as compared to the redox-insensitive micelles.

## 6. Summary and Future Outlook

It is becoming increasingly clear that nanocarriers will constitute an integral part of a therapeutic intervention in the future. The imagination of a chemist in designing and articulating their structures has paved the way for their translation into nanomedicine. The control offered by branched and hyperbranched structures in their design provides ample opportunities in placing any desired combination of functions into a single scaffold. These materials have offered great potential to develop materials with improved therapeutic efficacy including target specificity, controlled drug release, lower therapeutic doses and minimum exposure to normal tissues. This programmability of dendrimers and miktoarm stars is the key in developing highly efficient architectures with unique capabilities. The synthetic elaboration of dendrimers has reached a stage where one could easily adapt them to include chosen cores, branches and functional groups at surfaces. It provides freedom in incorporating a spatial distribution of diverse functionalities including therapeutic, targeting and imaging agents. With the ability to adapt to the needs of drug molecules, we can easily enable physical encapsulation or covalent linking and combinations thereof, in dendrimers and miktoarm stars. A hybrid combination of the latter two platforms may hold a better future, and a direction that can be explored. This review has highlighted the progress made in developing novel nanocarriers based on branched and hyperbranched architectures for nanomedicine. Much focus is currently being directed at taking the next step and bridging the gap to put these macromolecules into practice. Chemists are standing up to the challenge, and we are beginning to see translation of the structures elaborated by them, through a careful design and mix of adaptable features, into clinical studies. This will be achieved by synthetic adaptation of these nanostructures to industrial needs, knowledge of their safety, therapeutic efficacy, and a detailed understanding of their physico-chemical behavior.
